# Dopamine-Based Copolymer Bottlebrushes for Functional Adhesives: Synthesis, Characterization, and Applications in Surface Engineering of Antifouling Polyethylene

**DOI:** 10.1021/acsami.3c05124

**Published:** 2023-07-03

**Authors:** Roland Milatz, Joost Duvigneau, Gyula Julius Vancso

**Affiliations:** † Department of Materials Science and Technology of Polymers, and Department of Sustainable Polymer Chemistry, University of Twente, Enschede 7522 NB, The Netherlands; ‡ DPI, P.O. Box 902, 5600 AX Eindhoven, The Netherlands

**Keywords:** biomimetic adhesion, polydopamine, surface functionalization, polyethylene grafting, antifouling

## Abstract

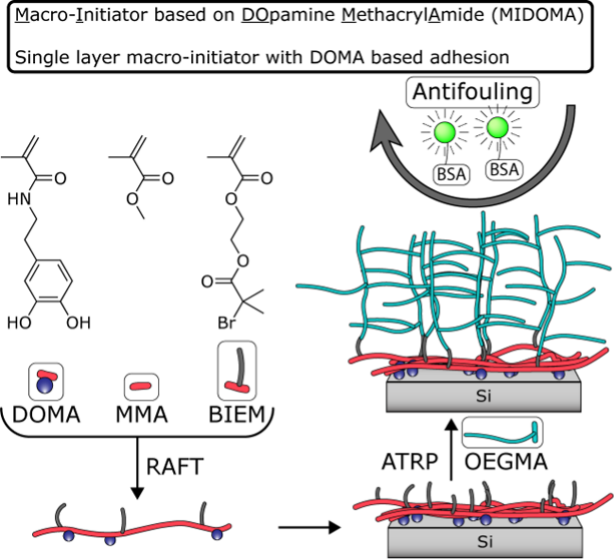

Nonpolar materials like polyolefins are notoriously challenging substrates for surface modification. However, this challenge is not observed in nature. Barnacle shells and mussels, for example, utilize catechol-based chemistry to fasten themselves onto all kinds of materials, such as boat hulls or plastic waste. Here, a design is proposed, synthesized, and demonstrated for a class of catechol-containing copolymers (terpolymers) for surface functionalization of polyolefins. Dopamine methacrylamide (DOMA), a catechol-containing monomer, is incorporated into a polymer chain together with methyl methacrylate (MMA) and 2-(2-bromoisobutyryloxy)ethyl methacrylate (BIEM). DOMA serves as adhesion points, BIEM provides functional sites for subsequent “grafting from” reactions, and MMA provides the possibility for concentration and conformation adjustment. First, the adhesive capabilities of DOMA are demonstrated by varying its content in the copolymer. Then, terpolymers are spin-coated on model Si substrates. Subsequently, the atom transfer initiator (ATRP) initiating group is used to graft a poly(methyl methacrylate) (PMMA) layer from the copolymers, with 40% DOMA content providing a coherent PMMA film. To demonstrate functionalization on a polyolefin substrate, the copolymer is spin-coated on high-density polyethylene (HDPE) substrates. A POEGMA layer is grafted from the ATRP initiator sites on the terpolymer chain on the HDPE films to provide antifouling characteristics. Static contact angle values and Fourier transform infrared (FTIR) spectra confirm the presence of POEGMA on the HDPE substrate. Finally, the anticipated antifouling functionality of grafted POEGMA is demonstrated by observing the inhibition of nonspecific adsorption of the fluorescein-modified bovine serum albumin (BSA) protein. The poly(oligoethylene glycol methacrylate) POEGMA layers grafted on 30% DOMA-containing copolymers on HDPE show optimal antifouling performance exhibiting a 95% reduction of BSA fluorescence compared to nonfunctionalized and surface-fouled polyethylene. These results demonstrate the successful utilization of catechol-based materials for functionalizing polyolefin surfaces.

## 1 Introduction

Adhesive fastening of different polymers in materials engineering is a grand challenge, particularly for nonpolar materials, such as polyolefins. In order to tackle the challenge of adhering to polyethylene, new adhesives, and methods need to be developed. One such method is “inspired” by nature to provide adhesives that possess properties paralleling bioadhesives, e.g., those utilized by barnacles.^1^ While the molecular principles of surface bonding have been elucidated both from the coordination chemistry^2^ as well as from the surface engineering^3^ points of view, there is still no synthetic generic adhesive system available based on catechols for widespread technological use.

In 2007, motivated by the protein mussels use for adhesion, a one-step method was described to coat objects of different materials, from metals to polyolefins.^4^ Mussels are known for their capability to adhere to various substrates and research has identified several chemical groups in their protein plaque essential for adhesion, including catechols and amines.^5^ Both groups can be found in the neurotransmitter dopamine (DA) species. Strongly adhering cross-linked polydopamine (PDA) films are deposited on many different substrates by submersion in an alkaline (pH ≥ 8.5) solution of DA. In addition, the adhesive bonding layer must also possess good cohesive strength for applications. To achieve cohesive strength, cross-linking is an attractive option. PDA films can be cross-linked through several different types of bonds.^6^

Once a film is bound to a substrate, other functional groups would expand the range of applications, e.g., by using these groups for subsequent surface functionalization. To tackle this challenge, we embarked upon an approach to combine catechols (PDA) and 2-(2-bromoisobutyryloxy)ethyl methacrylate (BIEM), which can serve as an initiator in subsequent atom transfer initiator (ATRP) polymerizations for the attachment of side groups on demand. Surface-initiated ATRP is an established technique for functionalizing different types of substrates, for example, flat wafers or particles.^7^

**Scheme 1:**
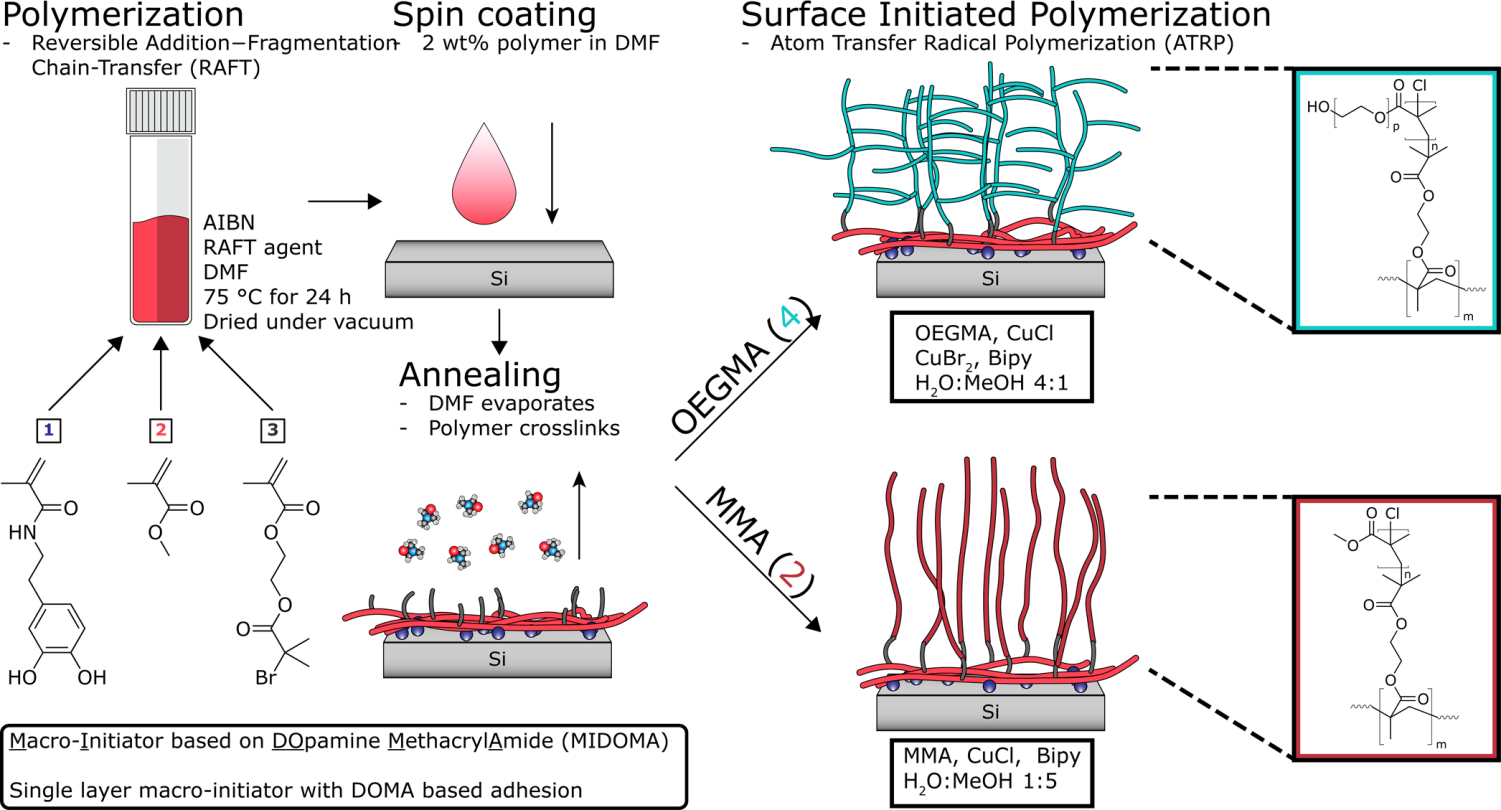
Schematic Representation of the Synthesis, Layer Application, and Grafting of a Polymer Layer from a Silicon Substrate Using MIDOMA as the Adhesive and Initiator Polymer is synthesized using RAFT polymerization of three monomers: DOMA (1), MMA (2), and BIEM (3). The polymer is spin-coated from a DMF solution and annealed overnight. Polymer (MMA (2) and OEGMA (4)) layers are grafted using surface-initiated ATRP. Si wafers are used as a model substrate but are later substituted for PE as the target substrate.

In this study, first, a DA-based monomer is synthesized.^8^ The monomer dopamine methacrylamide (DOMA) is copolymerized with 2-(2-bromoisobutyryloxy)ethyl methacrylate (BIEM), which provides functional Br sites for a subsequent ATRP grafting step. The subsequently grafted constituent can be chosen flexibly to allow for attachment to a variety of objects. The flexible choice of the grafted component ensures that we have adhesives for low and high surface energy substrates^9^ or functional adhesive layers, such as anticorrosive,^10,11^ antifogging,^12^ and antifouling^13−18^ layers, or surfactants for particle dispersion.^19^ In addition to the surface anchoring and the reactive side chain-linking groups, we also copolymerize a third inactive spacer component (methyl methacrylate (MMA)) to the functional terpolymer that provides a possibility to tune bottlebrush flexibility and functional group density. The components of the constituents of our designer adhesive bottlebrush terpolymer are shown in Scheme 1.

We synthesize DOMA-based copolymers^20^ with an atom transfer initiator (ATRP) functionality through reversible addition–fragmentation chain-transfer (RAFT) polymerization,^21^ as shown in Scheme 1. This copolymer is first applied onto clean silicon wafers to test the concept and then to square-shaped polyethylene (PE) pieces using spin coating to demonstrate the targeted antifouling application. From these substrates, we graft poly(methyl methacrylate)^22^ (PMMA) and poly(oligoethylene glycol methacrylate)^23^ (POEGMA) layers using ATRP. Here, POEGMA is used as a component to provide antifouling performance. We also show the effect of increasing catechol content on surface functionalization using contact angle, ellipsometry, and atomic force microscopy (AFM) measurements. To demonstrate the antifouling properties of POEGMA-decorated substrates, the nonspecific inhibition of fluorescein-labeled bovine serum albumin (BSA) protein adsorption is characterized with fluorescence microscopy. Fourier transform infrared (FTIR) spectroscopy is used to confirm and characterize the presence of the grafted layers on PE.

## 2 Experimental Section

### 2.1 Materials

2,2′-Bipyridyl (Reagentplus, ≥99%), 2-(2-bromoisobutyryloxy)ethyl methacrylate (BIEM, 95%), 4-cyano-4-(phenylcarbonothioylthio)pentanoic acid (RAFT agent, >97%), copper(I)chloride (CuCl, 97%), dibenzyl ether (purum, 98%), dopamine HCl, fluorescein-labeled bovine serum albumin (BSA-FITC), methacrylic anhydride (94%), methyl methacrylate (MMA, 99%), MgSO_4_ (ACS Reagent, ≥97%), oligoethylene glycol methacrylate (OEGMA, *M_n_* ∼500), phosphor buffered saline (PBS), polyethylene (high-density polyethylene (HDPE), pellets, product code 547999), sodium bicarbonate (≥99.7%), sodium hydroxide (NaOH, ≥98%), sodium tetraborate decahydrate (≥99.5%), and sulfuric acid (ACS Reagent, 95–97%) were bought from Sigma. CuCl was purified by cleaning 3× with acetic acid and subsequently 3× with ethanol. The PBS solution was prepared by dissolving one tablet in 200 mL of MilliQ water. CuBr_2_ (≥99%) was bought from Acros. Ethyl acetate (Analar Rectapur, 99.9%), *n*-hexane (GPR Rectapur, 98%), hydrochloric acid (35%), methanol (GPR Rectapur, 100%), and tetrahydrofuran (THF, GPR Rectapur, 100%) were purchased from VWR. Azobisisobutyronitrile (AIBN), dimethylformamide (DMF, Emsure), and hydrogen peroxide (H_2_O_2_, 30%) were obtained from Merck. AIBN was purified by recrystallization from methanol. MilliQ water was acquired using a MilliQ Advantage A10 purification system (Millipore, Billerica, Ma). Silicon wafers (100.0 ± 0.5 mm diameter and 525 ± 25 μm thickness, boron-doped with (100) orientation, 5–10 Ω·cm, Okmetic) were obtained from the Nanolab of the MESA + Institute of the University of Twente. HDPE square pieces were prepared by hot pressing HDPE pellets at 100 °C into larger flat substrates and cutting.

### 2.2 DOMA Synthesis

The dopamine methacrylamide synthesis is adapted using the procedures by Messersmith et al.^17^ with some modifications. Briefly, 5 g of sodium tetraborate decahydrate and 2 g of sodium bicarbonate are added to 50 mL of MilliQ water and stirred for 20 min under N_2_. 2 g (10.56 mmol) of dopamine HCl is added and the pH is increased to above 8 using a 1 M NaOH solution. 2 mL (13.43 mmol) of methacrylic anhydride is added to 20 mL of tetrahydrofuran. This solution is added dropwise to the aqueous solution. The combined solution is left to stir under N_2_ for 24 h after which the pH is reduced to below 2 using 1 M HCl. 50 mL of ethyl acetate is used three times to extract the monomer from the solution. Ethyl acetate is dried using MgSO_4_ and evaporated under a N_2_ stream to a brown slurry. This slurry is redissolved in 25 mL of ethyl acetate and precipitated in 200 mL of *n*-hexane. After decanting *n*-hexane, the last fraction of the solvent is evaporated under N_2_ and dried under vacuum at room temperature.

### 2.3 Copolymer Synthesis

Adapted from Yang et al.^20^ and Kafkopoulos et al.^21^ the copolymer of DOMA, MMA, and BIEM is synthesized using RAFT. The copolymer variations are named Dxx, where xx is the feed % of DOMA in the copolymer. Briefly, for D40, 9.9 mg (0.06 mmol) of azobisisobutyronitrile, 19 μL (0.1 mmol) of dibenzyl ether, 265.5 mg (1.2 mmol) of DOMA, 95.9 μL (0.9 mmol) of MMA, 192.8 μL (0.9 mmol) of BIEM, and 22.4 mg (0.08 mmol) of 4-cyano-4-(phenylcarbonothioylthio)pentanoic acid *N*-succinimidyl ester are dissolved in 3 mL of DMF. The solution is purged for 30 min under N_2_ and then placed under N_2_ overpressure into a 75 °C oil bath for 24 h. After this 24 h, the copolymer is precipitated in water and centrifuged for 30 min at 11,000 RPM. The solution is decanted and the polymer is dried for at least 72 h at 60 °C under vacuum. Before and after the reaction, the composition is determined using ^1^H NMR spectra measured on a 400 MHz Bruker AVANCE III AMX system (^1^H NMR spectra are given in the Supporting Information).

### 2.4 Film Formation

MIDOMA films are prepared by spin coating 2 wt % solutions in DMF on Si and HDPE substrates using a PI-KEM P6700 (Pi-KEM Limited) spincoater at 2000 rpm for 120 s. The substrates are then placed overnight in an oven at 60 °C under vacuum for annealing. After annealing, the substrates are rinsed with MilliQ water before rinsing with acetone and drying under N_2_.

### 2.5 Polymer Grafting

Both PMMA and POEGMA layers are grafted from MIDOMA using ATRP. The procedure for grafting PMMA is adapted from Yu et al.,^22^ whereas the procedure for POEGMA is adapted from Gunnewiek et al.^23^ Briefly, for PMMA, 10.64 purified MMA is added to 9 mL of solvent (methanol/water, 5:1) in a 50 mL round-bottomed flask and purged for 30 min with N_2_ under continuous stirring. In another round-bottomed flask, 312.4 mg (2 mmol) of bipyridine is added together with 99 mg (1 mmol) of CuCl and purged for 20 min with N_2_. Afterward, the solution of MMA is added to the flask with the catalyst and ligand and purged for 30 min again while stirring. The substrates spin-coated with the polymer are purged in a 50 mL Erlenmeyer flask for 30 min. The ATRP solution is then added to the Erlenmeyer flask containing the substrates and left to react for 24 h. Finally, the reaction is stopped by exposure to air and the substrates are removed and washed with copious amounts of water and then acetone and dried under N_2_.

For POEGMA brushes, 5 g of OEGMA and 163.4 mg of bipyridine are added to 9 mL of solvent (methanol/water, 1:4) and purged for 30 min in a small vial with a septum. In another vial, 37.5 mg of CuCl and 4 mg of CuBr_2_ are added and purged for 20 min with N_2_. The monomer- and ligand-containing solution is added to the vial containing the catalyst and purged for 30 min using N_2_. The substrates are placed in vials and purged with N_2_ for 30 min before the solution is divided between the vials containing the substrates. After the targeted duration of time (15, 30, or 60 min), the substrates are taken out and rinsed with copious amounts of water and ethanol and dried under N_2_. The naming convention for these samples is polymer type followed by MIDOMA composition, e.g., PMMA–D20, for PMMA grafted from D20.

### 2.6 Ellipsometry

Ellipsometry measurements are performed using an M-2000X spectroscopic ellipsometer (J.A. Woollam) with CompleteEASE, operating wavelengths from 245–1000 nm at three incident angles, 65, 70, 75°. For MIDOMA^24^ and POEGMA,^25^ a Cauchy model, *n* = *A* + *B*/λ^2^, with *A* = 1.45 and *B* = 0.01, is used for modeling the layer thickness. For PMMA,^26^*A* = 1.49 and *B* = 0. All measurements are done in air.

### 2.7 AFM

AFM measurements are performed on a Multimode 8 AFM instrument operated with a JV vertical engage scanner and a NanoScope V controller (Bruker). NCH cantilevers (Nanoworld, Switzerland) are used, with a spring constant of 42 N/m and a tip radius curvature of less than 8 nm. All AFM images are treated with the Flatten (0th order) and Plane Fit (1st order) calculations using NanoScope Analysis 2.0 software.

### 2.8 Contact Angle (Hysteresis)

Static and dynamic contact angles are measured using an OCA 15 device equipped with an electronic syringe unit (Dataphysics Instruments GmbH, Germany). MilliQ water is used as the probe liquid. At least 3 measurements per sample are performed and averaged.

### 2.9 FTIR

FTIR spectra are obtained with a Bruker α FTIR spectrometer equipped with a Platinum ATR single reflection crystal (Bruker Optic GmbH, Germany). Spectra are obtained between 4000-400 cm^–1^ with a resolution of 4 cm^–1^ at 64 scans.

### 2.10 Antifouling Assessment

2 mg of BSA-FITC is dissolved in 20 mL of PBS solution to obtain a 0.1 mg mL^–1^ solution. Substrates are incubated in 2 mL of solution for 3 h in the dark at 37 °C. FITC is characterized using a 460–490 nm excitation and 525 nm emission filter cube set. Images are processed using ImageJ software, version 1.53e.

## 3 Results and Discussion

### 3.1 MIDOMA Synthesis

First, we discuss the synthesis of a set of MIDOMA-based bottlebrush terpolymers. Each copolymer consists of three different monomers: (1) DOMA, (2) MMA, and (3) BIEM, as shown in Scheme 1. The concentrations of the constituents are varied as follows: for (1), between 0 and 40%; for (2), between 70 and 30% of the polymerization feed, respectively, such that BIEM is always 30% (% are molar concentrations). (1) is synthesized according to a modified procedure of Messersmith et al.^8^ The ^1^H NMR spectrum used for chemical identification can be found in Figure S1 in the Supporting Information. Polymerization is performed using a RAFT protocol adapted from Kafkopoulos et al.^21^ Composition and conversion are measured using ^1^H NMR during and after the polymerization, as shown in Figure S2 in the Supporting Information. Shown in Figure S3 is a ^1^H DOSY NMR spectrum of MIDOMA with 40% DOMA. The presence of all three monomers around the same diffusion coefficient values proves the successful synthesis of a copolymer. The naming of and referrals to the MIDOMA copolymers are derived from the percentage of (1) in the feed content, e.g., D40 for 40% DOMA.

### 3.2 MIDOMA Spin Coating

The terpolymers are applied on a substrate by spin coating. We use Si wafers and HDPE square pieces as substrates for film formation. The size of each substrate is ∼1 × 1 cm^2^. The polymers are first dissolved at 2 wt % in DMF and then spin-coated. After spin coating, they are shortly annealed to compact the layer and remove any residual solvent. Finally, they are washed to remove any physisorbed polymer residue.

**Figure 1:**
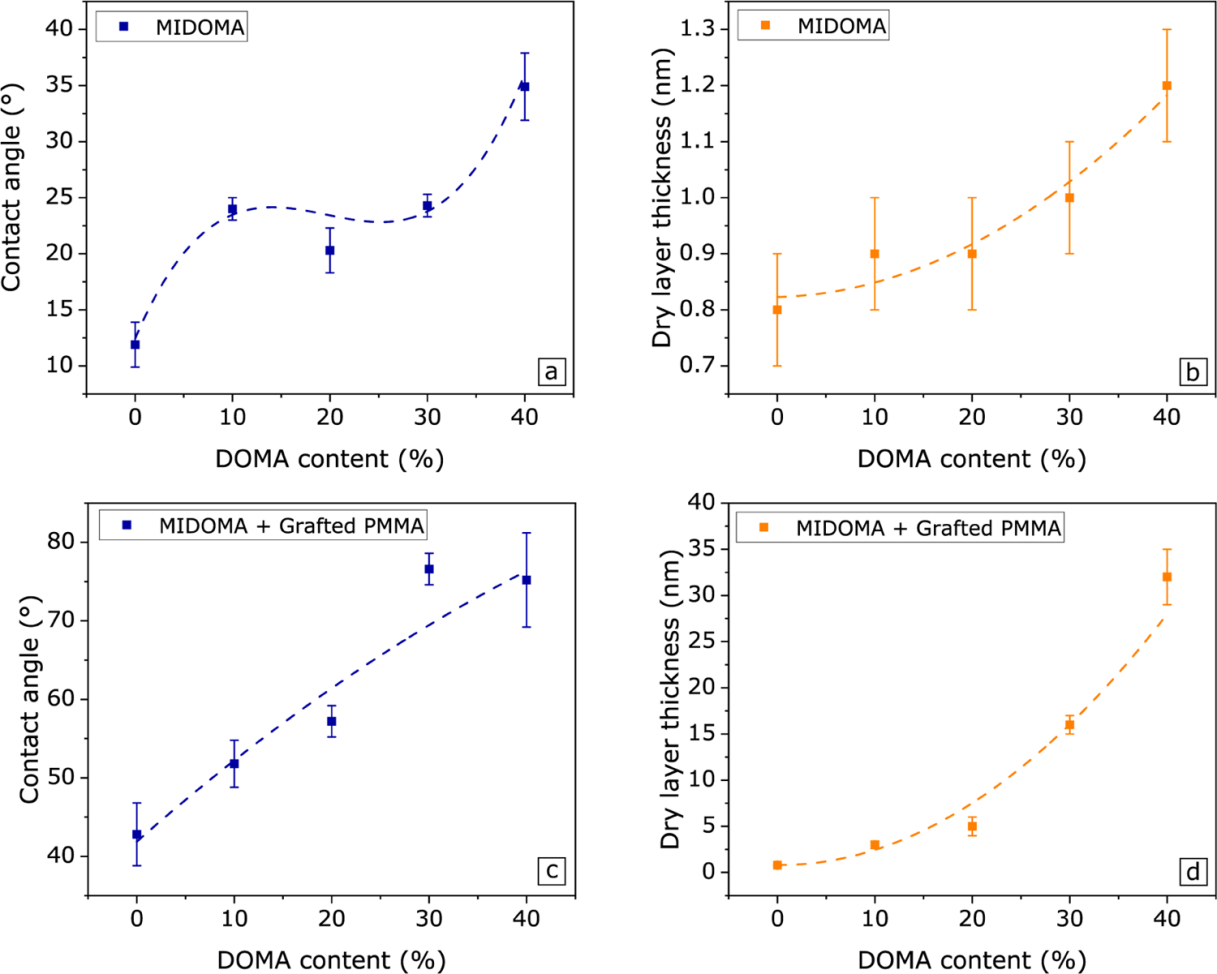
Static contact angle (a, c) and film dry thickness (b, d) of MIDOMA and MIDOMA + grafted PMMA layers, respectively, as a function of DOMA content. The dashed lines are a guide to the eye. For the contact angle, D40 and D30 have the highest angle for MIDOMA in panel (a) and PMMA-grafted layer in panel (c), respectively. D40 has the highest layer thickness for both the MIDOMA and PMMA-grafted layers in panels (b, d).

Before grafting a PMMA layer from the BIEM moiety, the static contact angle and dry layer thickness are measured for each MIDOMA composition; the results are shown in Figure 1a,b. Raw data on the contact angle and dry layer thickness values can be found in Tables S1, S2, and S3. We see for both the contact angle values, as well as for the layer thickness values, that a minimum thickness value is obtained for D0 and the maximum for D40. However, the trends do not follow a linear behavior: between D10 to D30, a plateau is reached. Here, each copolymer possesses similar contact angle and layer thickness values. By comparing Table S1 and Figure 1a, we can see that through the decrease in contact angles, most of the physisorbed material is removed. This indicates that complete surface coverage is not reached because the pure material (i.e., MIDOMA) has a contact angle value close to that of PMMA. Thus, part of the hydrophilic silicon wafer surface is still accessible to the water droplet.

### 3.3 Polymer Grafting by ATRP

Subsequently, a PMMA layer is grafted from each MIDOMA layer using ATRP according to procedures described in the literature.^22,23^ The choice of catalysts and ligands is shown in Scheme 1. MIDOMA-coated substrates from which polymers have been grafted are subsequently referred to as PMMA–Dxx or POEGMA–Dxx, for the different polymer layers of PMMA and POEGMA, respectively. The static contact angles and dry layer thickness of these grafted layers are shown in Figure 1c,d. Here, we see different behavior for each MIDOMA layer. PMMA–D0 does not have any appreciable increase in thickness, nonetheless, the related contact angle increases. For each PMMA–MIDOMA composition, the static contact angle also increases, but the maximum is reached for PMMA–D30. The contact angle of both PMMA–D30 and PMMA–D40 layers are in the same range as the static contact angle for bulk PMMA. However, the PMMA–D30 layer is only 10 nm thicker than PMMA–D20, at 16 nm, while the PMMA–D40 layer is 17 nm thicker than that of PMMA–D30. While the contact angle measurements suggest otherwise, the surface morphology between PMMA–D30 and PMMA–D40 must be different. Assuming similar grafted chain lengths, the grafting density must therefore increase for higher DOMA content.

Contact angle hysteresis measurements are performed on PMMA–D30 and PMMA–D40 with grafted PMMA layers, and the data are shown in Table 1. Here, we find that neither PMMA–D30 nor PMMA–D40 exhibit any appreciable difference in contact angle hysteretic behavior from bulk PMMA as reported in the literature.^27^

**Table 1:** Advancing and Receding Contact Angles for the Grafted PMMA Layer on MIDOMA with 30 and 40% DOMA Content^a^

	θ_Adv_ (°)	θ_Rec_ (°)	hysteresis (°)
PMMA–D30	78 ± 2	37 ± 1	41 ± 2
PMMA–D40	81 ± 2	33 ± 1	48 ± 2

a Literature: θ_Adv_: 78 ± 2° θ_Rec_: 33 ± 5° Hysteresis: 45 ± 5°.^21^

### 3.4 Surface Characterization by AFM

**Figure 2:**
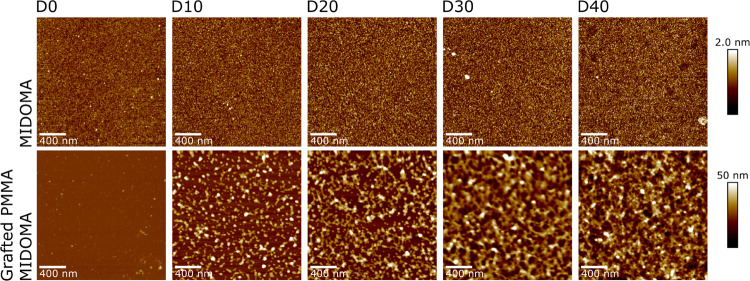
AFM height maps of MIDOMA (top) and MIDOMA + grafted PMMA (bottom) layers as a function of increasing DOMA content (left to right). As the DOMA content increases, the MIDOMA layer’s roughness increases and the grafted PMMA layer changes from disconnected blobs (PMMA–D10) to a network (PMMA–D30) and a full layer (PMMA–D40).

The surface morphology of the substrates is characterized by AFM in tapping mode for both the MIDOMA and PMMA-grafted layers. Representative images are captured in Figure 2. It can be seen that the very thin MIDOMA layers increase in roughness (the RMS roughness, *R*_q_, values increase from 0.2 to 0.3 nm, for D0 and D40, respectively) as the DOMA content increases. However, the appearance of the surface structures remains essentially unchanged. In contrast, the grafted PMMA layers show significant changes in the surface structure with increasing DOMA content in the copolymer. The surface of the grafted layer grown from PMMA–D0 shows essentially no difference when compared with the MIDOMA layer. On the contrary, for PMMA–D10 and PMMA–D20, many polymeric blobs (we propose that these features correspond to chain sections collapsed upon themselves) are present on a flat silicon surface, with typical dimensions at the nanoscale. As the DOMA concentration, thus the adhesive content, increases, the surface blob structures form networks and at PMMA–D30 seems to form a connected “two-dimensional (2D)” network, covering the entire surface.

**Figure 3:**
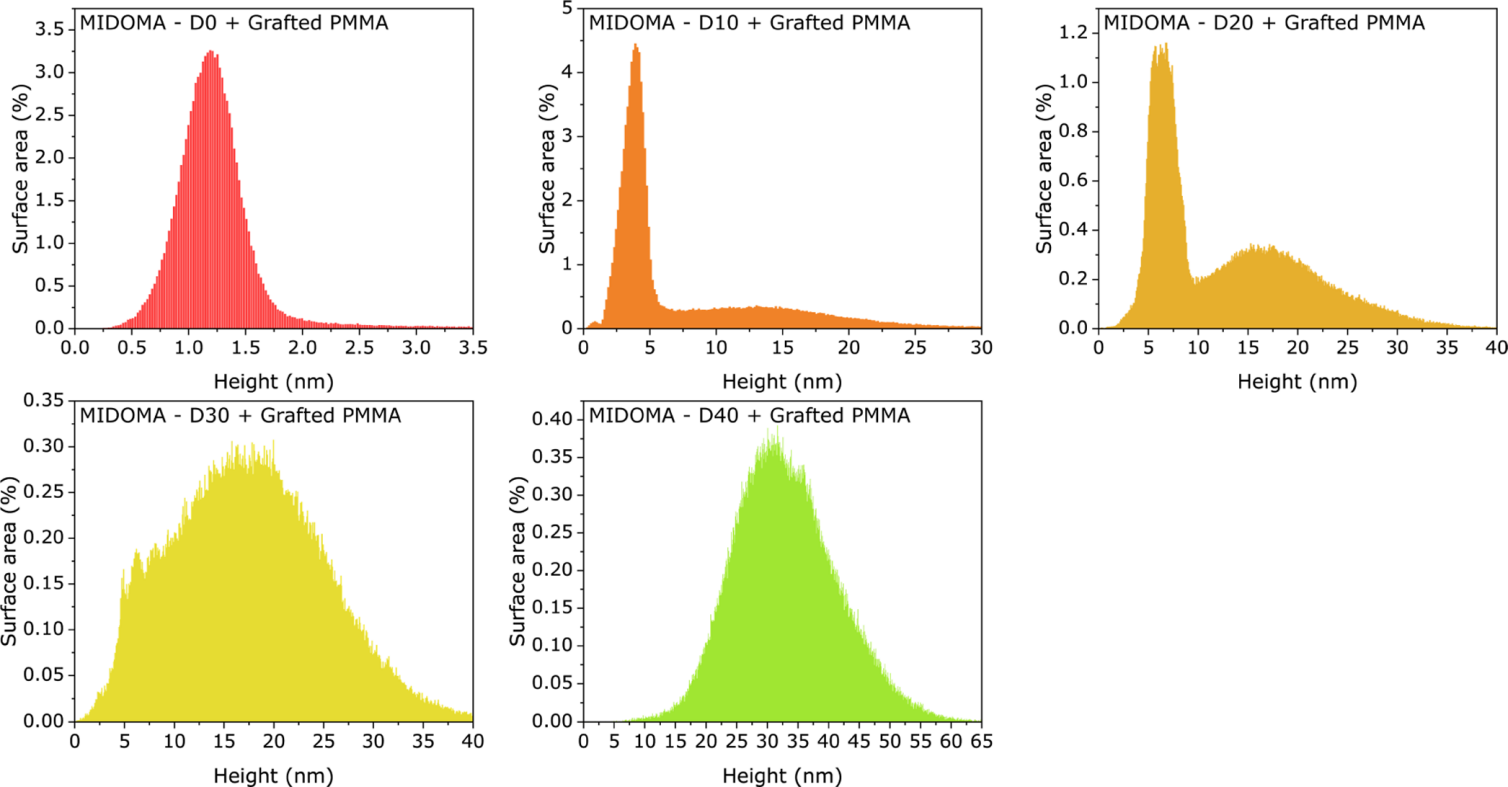
Height histograms for PMMA–D0 to PMMA–D40. (a) PMMA–D0, (b) PMMA–D10, (c) PMMA–D20, (d) PMMA–D30, and (e) PMMA–D40. Bimodal distributions are visible for PMMA–D10 to PMMA–D30 DOMA concentrations.

In Figure 3, we show height histograms calculated from the bottom row of the images shown in Figure 2. In general, two different distributions are visible, monomodal and bimodal, with different maximum values. For PMMA–D0, we see a narrow monomodal distribution with a maximum value at 1.2 nm height. We suggest that this distribution is related to the roughness of the substrate combined with tightly bound polymer chains at the surface (without loops or blobs). PMMA–D10 and PMMA–D20 exhibit bimodal height distributions with a pronounced sharp constituent at low height values, and broad contributions with maxima at 15 nm (PMMA–D10; Figure 3b) and 17 nm (PMMA–D20; Figure 3c), respectively. We assign the sharp distributions again to the combined roughness contributions of the Si substrate and to tightly bound chains. The broader components then are assigned to contributions of the increasing amount of blobs. For specimens PMMA–D30 and PMMA–D40, the distributions are dominated by contributions of the blobs. Thus, we see a diminishing contribution of the Si substrate (combined with the flat-lying, tightly bound chains) to the histograms with increasing DOMA concentration. We interpret this observation as a sign of forming increasingly coherent films at high relative DOMA values.

**Figure 4:**
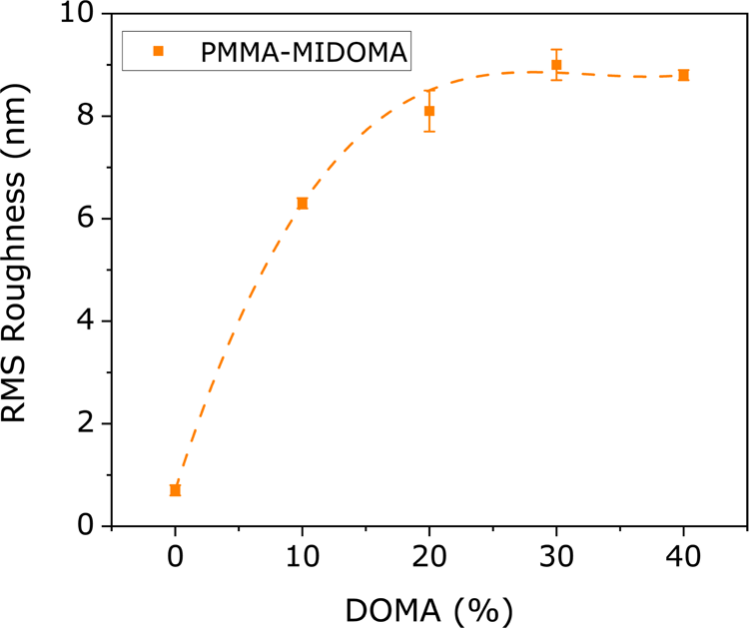
Root-mean-squared (RMS) roughness values calculated for PMMA–MIDOMA from AFM height maps with scan sizes of 5 μm.

The difference in surface coverage between PMMA–D30 and PMMA–D40 is assumed to be caused by the density of BIEM groups on the surface. Higher surface coverage means that the BIEM density is higher and thus PMMA chains are grafted closer together. The steric repulsion among polymer chains will push them in the only free direction, i.e., normal to the surface, leading to thicker PMMA layers (see also Scheme 1). From these images, the surface roughness, *R*_q_, can be calculated, as shown in Figure 4. While PMMA–D10 and upward show higher roughness, PMMA–D0 shows very low roughness values. This low *R*_q_ indicates that the surface essentially features only tightly adsorbed macromolecules.

In conclusion for this section, we demonstrate the possibility to graft a PMMA layer on top of a macroinitiator on silicon wafers. To completely cover the surface, at least 40% DOMA is needed in the macroinitiator.

**Figure 5:**
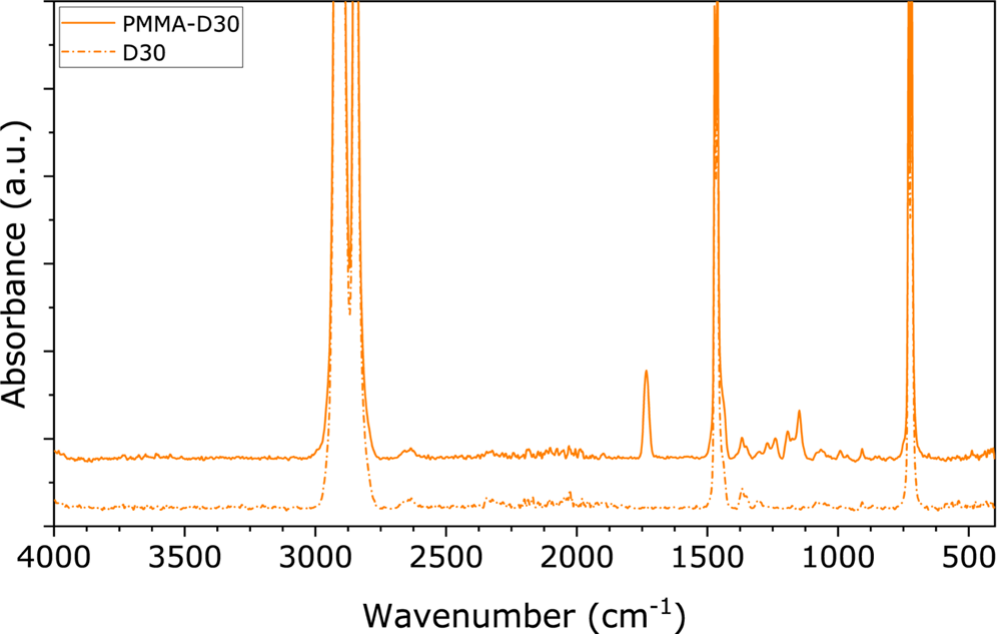
FTIR absorption spectrum of D30 (solid) and PMMA–D30 (dashed) on PE. Functional groups present are as follows: −CH_2_– backbone at ∼2990 cm^–1^, C=O ester at ∼1750 cm^–1^, −O–CO–CH_3_ ester group at ∼1400 cm^–1^, −C–O– bond at ∼1300–1100 cm^–1^.

The next step is to apply and assess the adhesion of our macroinitiators on the HDPE substrates. In Figure 5, ATR-FTIR spectra before and after the grafting of PMMA from the MIDOMA coating to HDPE are shown. The dashed line indicates very low concentrations of the MIDOMA coating before polymer grafting. These layers are too thin to be captured by these spectra. However, the FTIR spectra clearly prove the presence of grafted PMMA chains. In conclusion, we demonstrate that it is possible to graft polymer layers from the HDPE surfaces using our MIDOMA coatings.

### 3.5 Polymer Grafting for Anti-Biofouling Functionality

**Figure 6:**
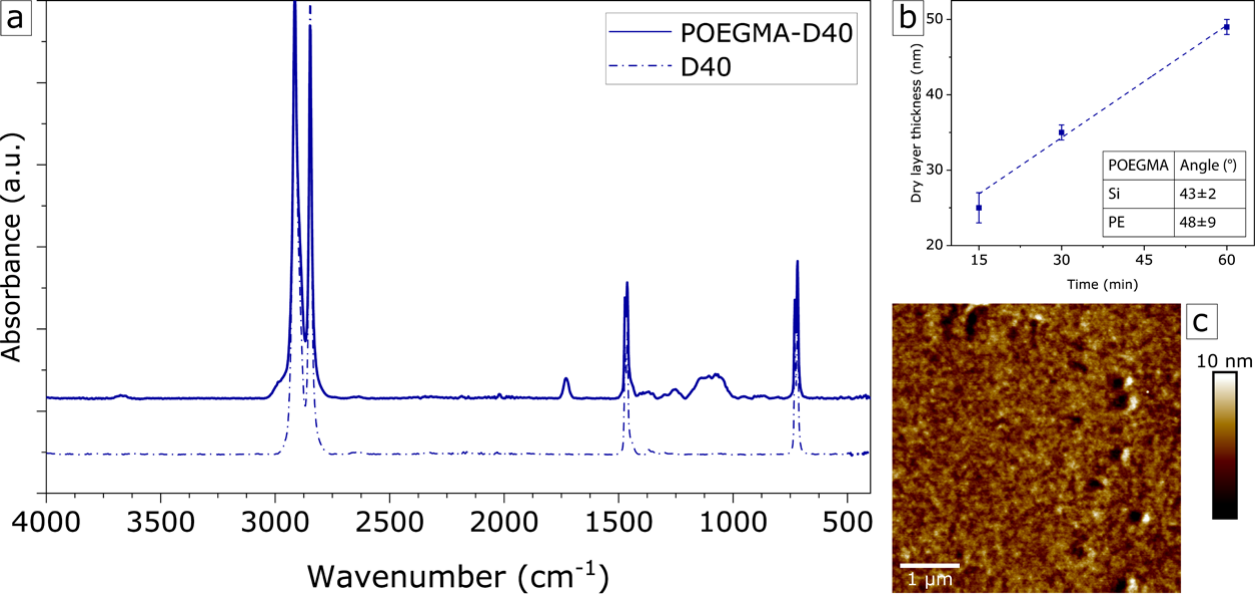
(a) FTIR spectrum of D40 (dashed) and POEGMA–D40 (solid) on HDPE. Functional groups present are as follows: −CH_2_– backbone at ∼2990 cm^–1^, C=O ester at ∼1750 cm^–1^, −O–H ethylene glycol chain end at ∼1410 cm^–1^, −C–O– ethylene glycol chain at ∼1250–1000 cm^–1^. (b) Grafted POEGMA film dry thickness (nm) as a function of polymerization time (min). Inset: The static contact angle values for POEGMA on Si and PE. (c) AFM height map of MIDOMA–D40 + grafted POEGMA.

In the next step, we show the possibility of using a MIDOMA layer as the platform for creating a functional, anti-biofouling coating. D40, from which, the best PMMA layer can be grafted, is used as the starting material. An established material in the field of antifouling, poly(ethylene glycol), is used as a methacrylate end-capped monomer (oligoethylene glycol methacrylate, OEGMA) for demonstrating antifouling properties. A bottlebrush type of polymer is prepared by ATRP from a surface-adhering MIDOMA layer. First, we tackle the question of film thickness development with polymerization time. To this end, we first employed silicon wafer substrates, from which the POEGMA layer was grafted. In Figure 6b, we show the layer thickness as a function of polymerization time. A thickness value of approx. 50 nm is reached at 60 min, which we consider a good value. We mention that for longer polymerization times, the surface layer begins to gel, which should be avoided. The homogeneity of the grafted layer is also best for this sample, as observed by the eye.

POEGMA is then also grafted from MIDOMA-coated HDPE surfaces. We assume here that the thickness values of these films are essentially the same as the layers grown on TiO_2_ over the same polymerization time (POEGMA layer thicknesses on TiO_2_ substrates as determined by AFM step height measurements are shown in Figure S5). Static contact angles measured on these substrates are summarized in the inset in Figure 6b. Both coated Si and HDPE surfaces possess a contact angle value close to that of values reported for POEGMA layers in the literature.^25^ The large standard deviation on HDPE suggests that these surfaces are less homogeneous.

ATR-FTIR spectra were also obtained for these HDPE substrates, as shown in Figure 6a. In the spectrum of grafted POEGMA, several peaks are visible, corresponding to the methacrylate ester group as well as the ethylene glycol group and −OH end group at 1750, 1410, and 1250–1000 cm^–1^, respectively. Coupled with the presence of the −CH_2_– backbone, the successful grafting of a POEGMA layer from MIDOMA on the HDPE is thus confirmed.

Shown in Figure 6c is an AFM height map of a POEGMA layer grafted from MIDOMA–D40. For POEGMA or similar ethylene glycol materials, the antifouling functionality is based on forming a hydration layer.^17^ This is a layer of tightly bound water on the POEGMA layer, which prevents the binding of proteins or other biomaterials to the surface. For strong antifouling properties, a smooth, homogeneous layer is desired.^28^ As shown in Figure 6c, we have obtained a smooth POEGMA layer.

### 3.6 Nonspecific Inhibition of BSA Protein Adsorption

**Figure 7:**
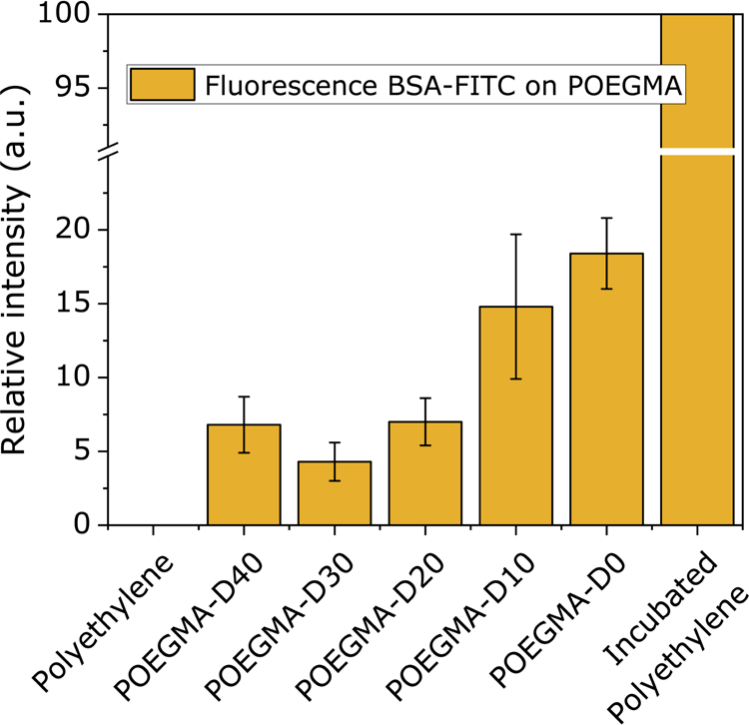
Relative intensity of fluorescent emission by BSA-FITC adsorbed on each substrate. Normalized such that incubated, unmodified HDPE is 100% and unincubated, unmodified HDPE is 0%. Images were converted to the RGB stack and the value for G was used for calculating these values.

Anti-biofouling is tested on POEGMA grafted from PE. Specifically, the nonspecific inhibition of the adsorption of proteins is probed by incubation in a BSA-FITC solution, where FITC provides fluorescence. The fluorescent behavior of these incubated substrates was tested using fluorescence microscopy. Shown in Figure S6 in the Supporting Information are example images from each set of substrates. An unmodified HDPE substrate was incubated as the 100% reference. A clean, unmodified HDPE substrate was measured, but not incubated, to serve as the 0% reference. The other images were normalized with regard to these two points and the data has been plotted in Figure 7.

First, as shown in Figure 7, POEGMA–D30 gives the best antifouling performance at <5% relative intensity. Second, POEGMA–D20 has an antifouling performance similar to POEGMA–D40 of below 7.5%. Finally, from POEGMA–D10 to POEGMA–D20, the fouling is more than halved from 15 to 7.5%. In Figure S6, the comparison between layer thickness and antifouling is shown. Here, we can see that the POEGMA layers are 33 and 50% thinner for D10 and D0, respectively, compared to D30. However, the fouling is almost 3 to 4 times higher. In all cases, however, the grafted POEGMA coating substantially improves the antifouling behavior compared to unmodified PE.

## 4 Conclusions

We describe the successful synthesis and characterization of a three-component, bio-inspired molecular “primer coating” with polydopamine functional units for surface attachment, spacers for coverage control, and functional groups for subsequent attachment of side chains. A thorough characterization of the molecular structure is provided. Films of the terpolymer are fabricated by spin coating the macroinitiator on hydrophilic silicon wafers and hydrophobic polyethylene. Through characterization of these layers using ellipsometry and AFM, we show that the formed MIDOMA layers are very thin; essentially, the thickness is independent of the DOMA content of the macroinitiator. Nonetheless, by grafting PMMA layers from the functional film, the importance of the right adhesive content in the terpolymer is demonstrated. The grafted layers can become several tens of nanometers thicker with increasing amounts of DOMA. We also show the presence of PMMA layers grafted from the HDPE surfaces using FTIR. We demonstrate that using POEGMA grafted from our MIDOMA layer, the nonspecific adsorption of fluorescein-labeled BSA can be reduced by more than 90% compared to an unmodified HDPE substrate. To summarize, we demonstrate using a PDA-derived copolymer to introduce functionalities to hydrophilic silicon and hydrophobic HDPE surfaces.

## Supporting Information Available

^1^H NMR spectra of DOMA and MIDOMA; FTIR spectra of MIDOMA-coated HDPE films; fluorescence microscopy images; and ellipsometry and contact angle data are provided (PDF)
