# Supplementary material for: Dopamine-Based Copolymer Bottlebrushes for Functional Adhesives: Synthesis, Characterization, and Applications in Surface Engineering of Antifouling Polyethylene
Source: ACS Appl Mater Interfaces. 2023 Jul 3;15(28):34023–30. doi: 10.1021/acsami.3c05124 (PMC10360033; doi:10.1021/acsami.3c05124)
Supplement: Supplementary file 1 — am3c05124_si_001.pdf [file am3c05124_si_001.pdf]

Supporting Information

Dopamine-based copolymer bottlebrushes for functional adhesives: Synthesis, characterization and applications in surface engineering of antifouling polyethylene

Roland Milatz,[†,‡] Joost Duvigneau*,[†] and G. Julius Vancso*,[†]

†Department of Materials Science and Technology of Polymers, University of Twente, Enschede 7522 NB, the Netherlands.

‡DPI, P.O. Box 902, 5600 AX Eindhoven, the Netherlands.

Corresponding Authors

*E-mail: g.j.vancso@utwente.nl (G.J.V.).

*E-mail: j.duvigneau@utwente.nl (J.D.).

SI.1 ^{1}H-NMR of DOMA and MIDOMA

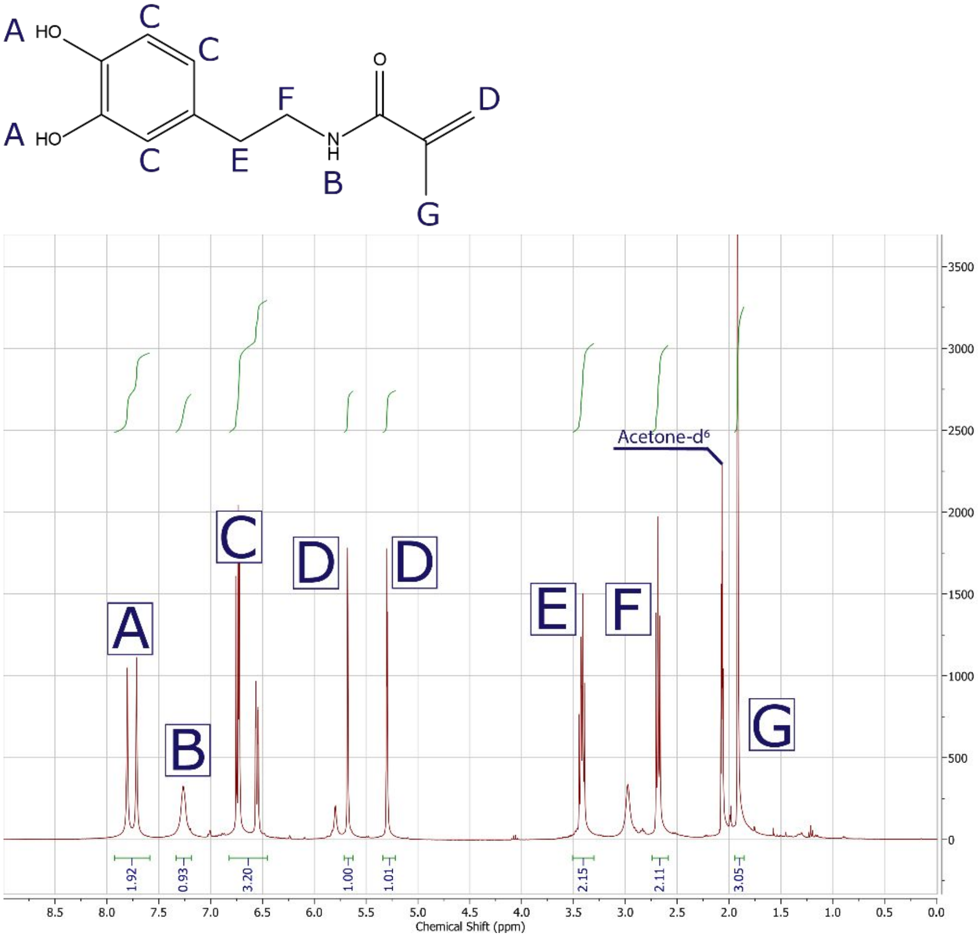

Figure S1 ^{1}H-NMR spectrum of dopamine methacrylamide monomer in acetone-d6. All peaks are marked with letters corresponding to the protons in the image above.

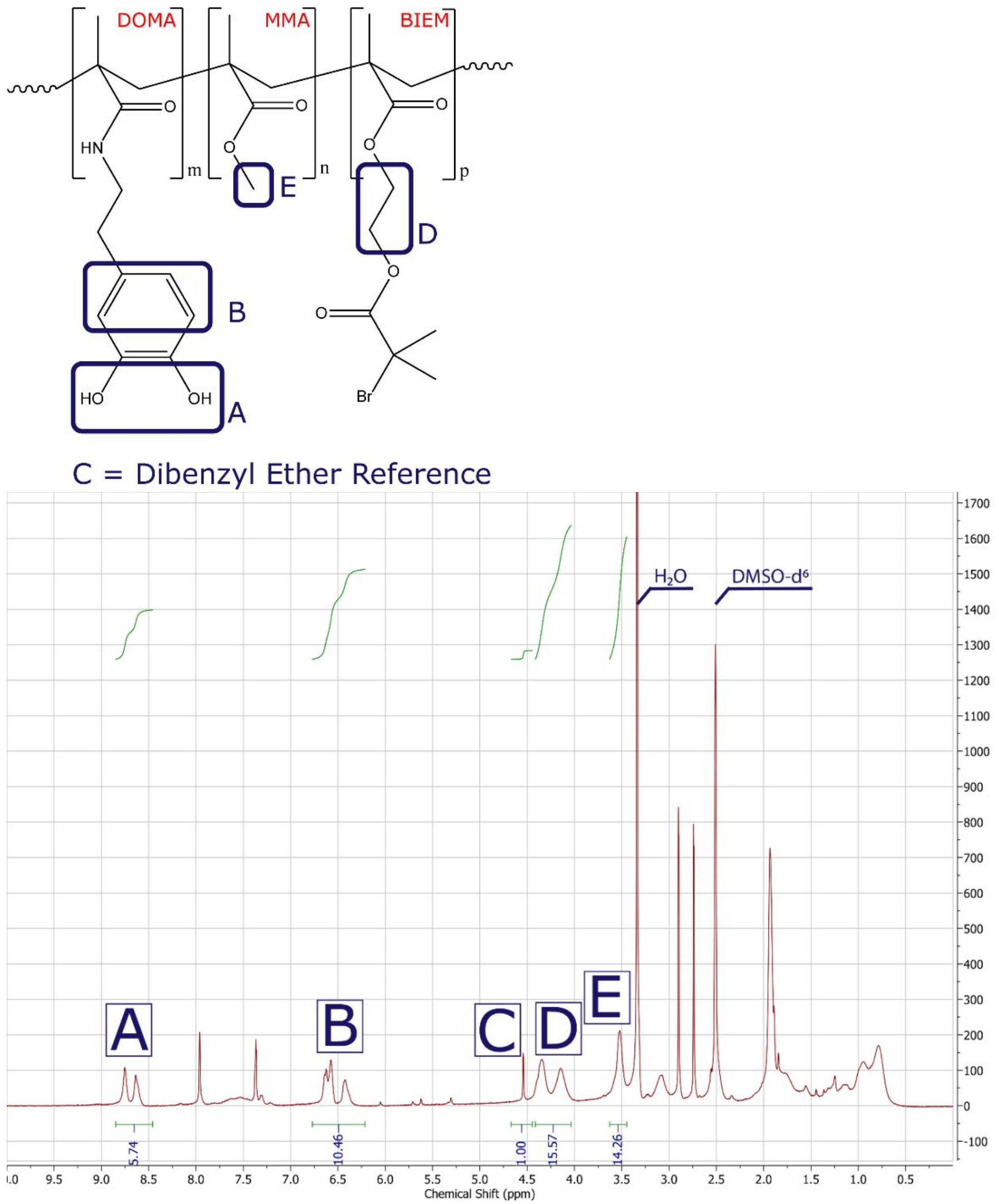

Figure S2 ^{1}H-NMR spectrum of MIDOMA copolymer in DMSO-d6. All peaks are marked with letters corresponding to the protons in the image above.

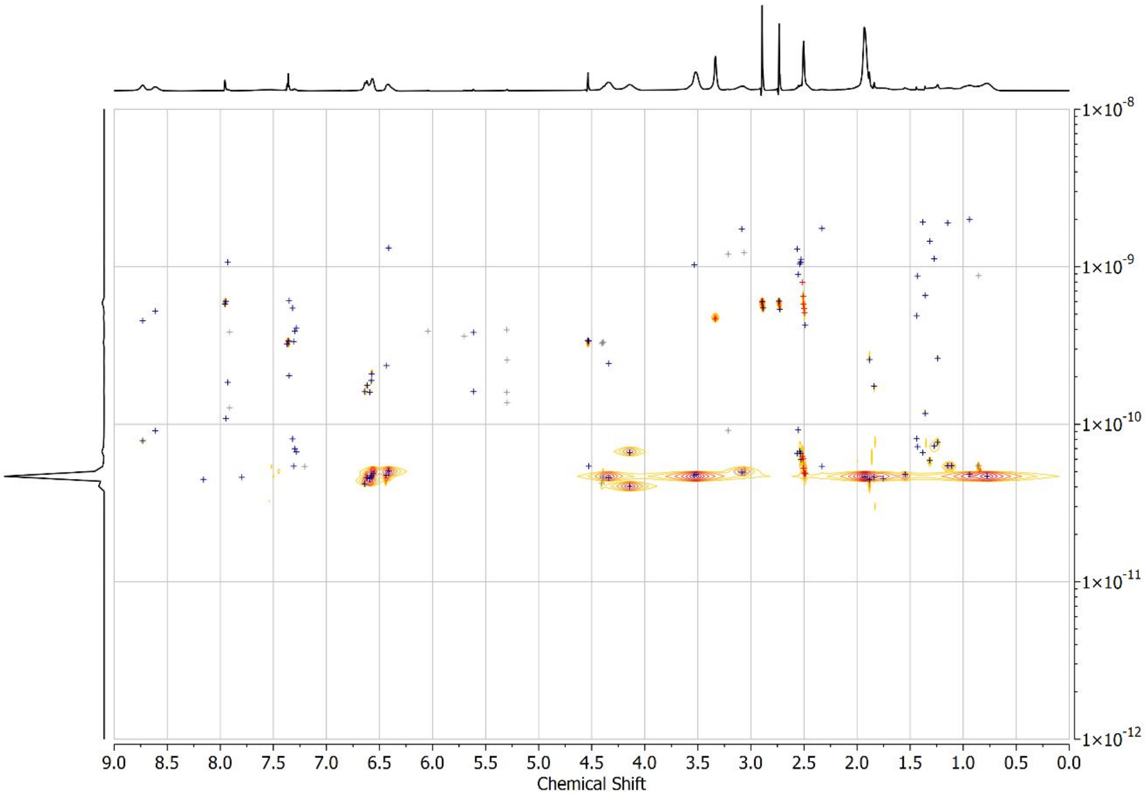

Figure S3 ^{1}H DOSY NMR spectrum of MIDOMA copolymer in DMSO-d6. The peaks corresponding to the different monomers all possess similar diffusion values, showing we have a copolymer.

SI.2 FTIR of MIDOMA-coated PE films

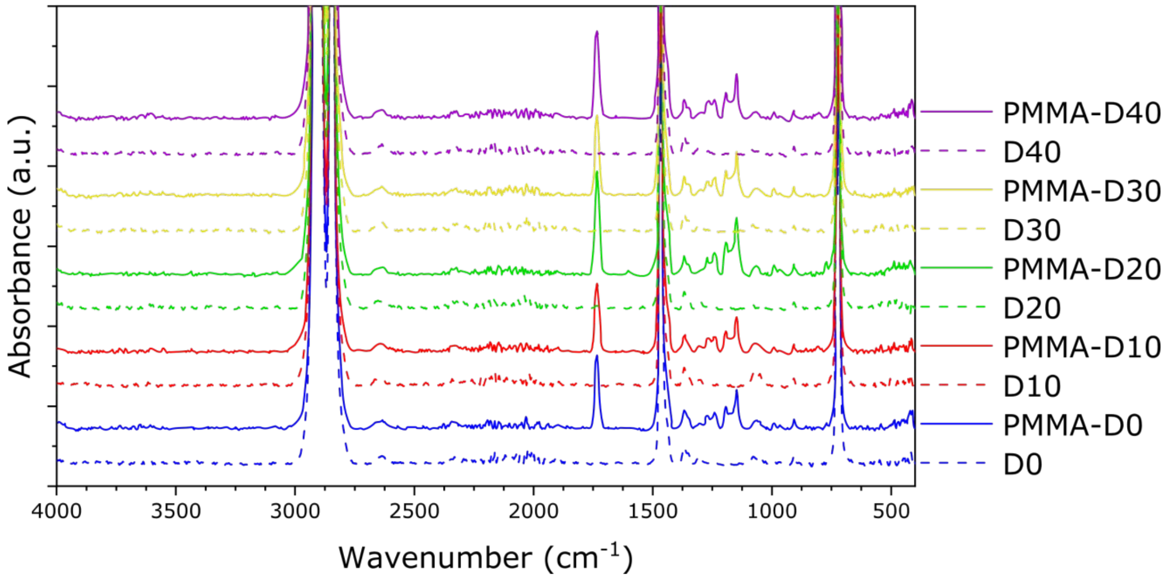

Figure S4 FTIR absorption spectra for all MIDOMA compositions with and without a grafted PMMA layer.

SI.3 Fluorescence Microscopy

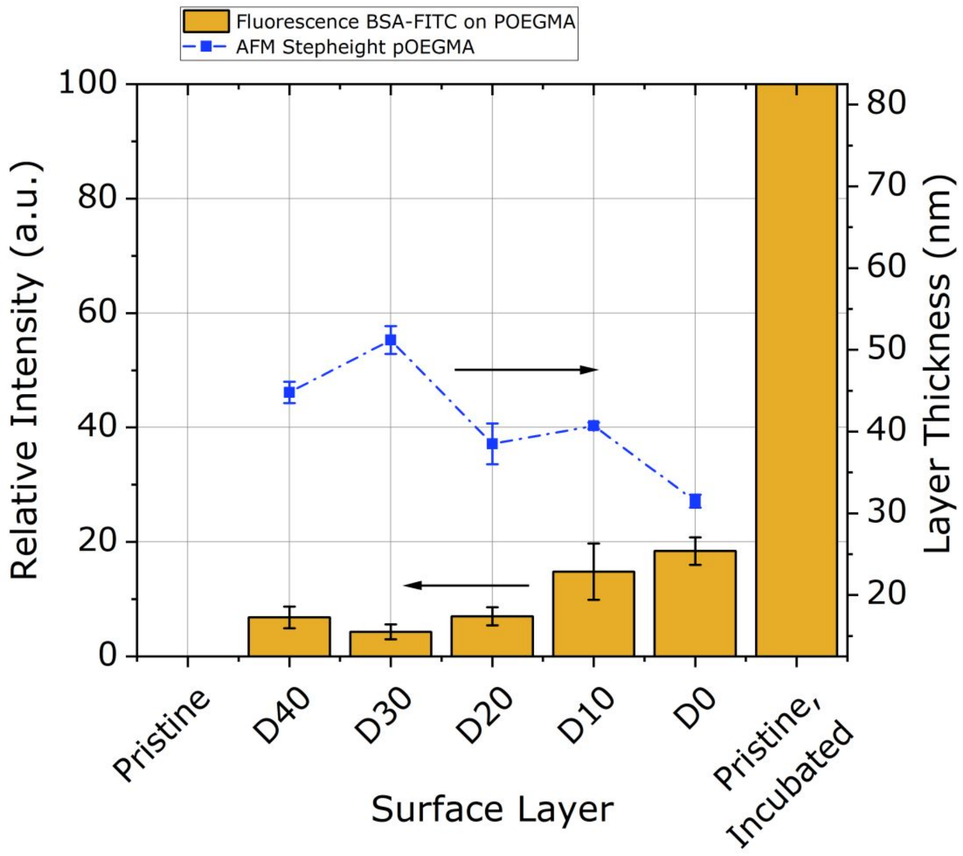

Figure S5 Relative intensity of fluorescent emission by BSA-FITC adsorbed on each substrate with the corresponding thickness of the POEGMA layer as measured on TiO_2 substrates. Normalized such that incubated, unmodified HDPE is 100% and unincubated, unmodified HDPE is 0%. Images were converted to RGB stack and the value for G was used for calculating these values.

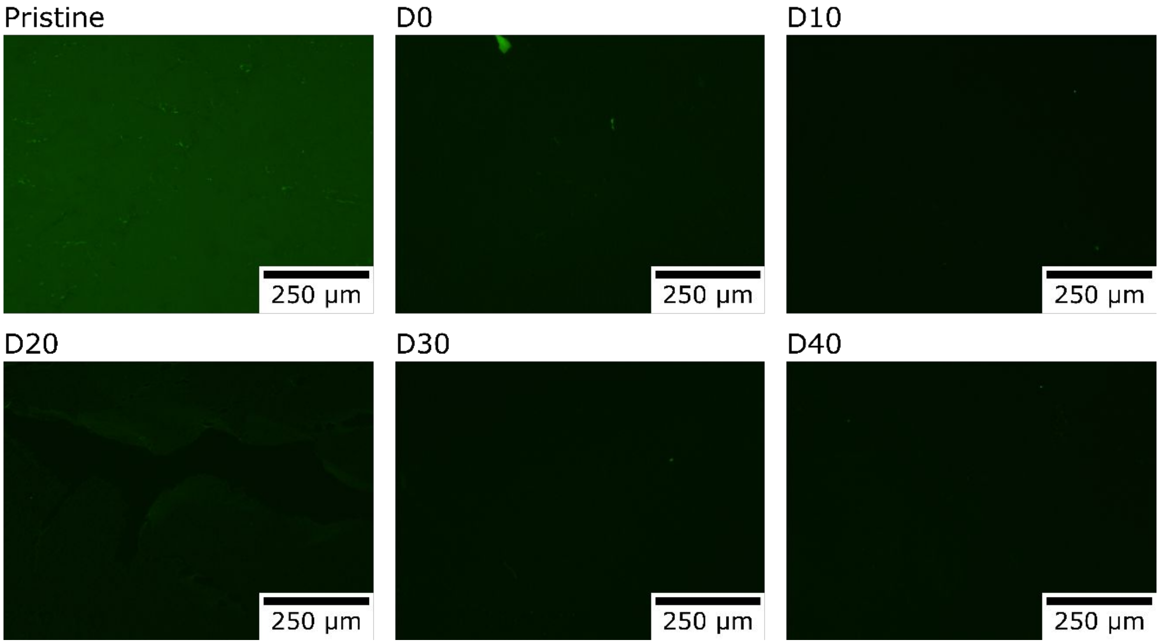

Figure S6 Fluorescence microscopy images of fluorescent protein on POEGMA layers on MIDOMA and pristine HDPE. Brightness has been increased by 50% for viewing only.

SI.4 Ellipsometry and Contact Angle

Ellipsometry fitting: n = 1.49 (Literature value for PMMA), no other fitting parameters were used. POEGMA: $n = A+B/\lambda^2$ with A = 1.45 and B = 0.01.

DOMA content	Contact Angle (°)
**0**	65.5 ± 1
**10**	73.6 ± 1
**20**	72.9 ± 1
**30**	72.4 ± 1
**40**	72.0 ± 0

Table S1 Contact angles of MIDOMA spin coated on Si wafers after annealing, but before the washing step.

DOMA content	Contact Angle (°)	Layer Thickness (nm)
**0**	11.9 ± 2	0.8 ± 0.1
**10**	24.0 ± 1	0.9 ± 0.1
**20**	20.3 ± 2	0.9 ± 0.1
**30**	24.3 ± 1	1 ± 0.1
**40**	34.9 ± 3	1.2 ± 0.1

Table S2 Contact angles and ellipsometry data from **Figure 1**, after annealing and washing.

DOMA content	Contact Angle (°)	Layer Thickness (nm)
**0**	42.8 ± 4	0.8 ± 0.1
**10**	51.8 ± 3	3.5 ± 0.4
**20**	57.2 ± 2	5.2 ± 1
**30**	76.6 ± 2	16 ± 1
**40**	75.2 ± 6	32 ± 3

Table S3 Contact angles and ellipsometry data from **Figure 1**, after grafting PMMA from the spin coated MIDOMA layers.

Time (minutes)	Average (nm)
**15 minutes**	25 ± 2
**30**	35 ± 1
**60**	49 ± 1

Table S4 Ellipsometry data from **Figure 6**, after grafting POEGMA from the spin coated D40 layers.

POEGMA	Angle(°)
**Si**	42.8±2
**PE**	48.0±9

Table S5 Contact angle data of POEGMA on Si and PE substrates.
